# BfmRS encodes a regulatory system involved in light signal transduction modulating motility and desiccation tolerance in the human pathogen *Acinetobacter baumannii*

**DOI:** 10.1038/s41598-022-26314-8

**Published:** 2023-01-05

**Authors:** Bárbara Perez Mora, Rocío Giordano, Valentín Permingeat, Malena Calderone, Natalia Arana, Gabriela Müller, Ramiro E. Rodríguez, Renatas Krasauskas, María Alejandra Mussi

**Affiliations:** 1 Centro de Estudios Fotosintéticos y Bioquímicos (CEFOBI-CONICET), Universidad Nacional de Rosario (UNR), Rosario, Argentina; 2 Instituto de Biología Molecular y Celular de Rosario (IBR-CONICET), Universidad Nacional de Rosario, Rosario, Argentina; 3 Centro de Estudios Interdisciplinarios, Universidad Nacional de Rosario, 2000 Rosario, Argentina; 4 Institute of Biosciences, Life Sciences Center, Vilnius University, Vilnius, Lithuania; 5 Institute of Biotechnology, Life Sciences Center, Vilnius University, Vilnius, Lithuania

**Keywords:** Genetics, Microbiology

## Abstract

We have previously shown that *Acinetobacter baumannii* as well as other relevant clinical bacterial pathogens such as *Staphylococcus aureus* and *Pseudomonas aeruginosa*, perceive and respond to light at 37 °C, the normal temperature in mammal hosts. In this work, we present evidence indicating that the two-component system BfmRS transduces a light signal in *A. baumannii* at this temperature, showing selective involvement of the BfmR and BfmS components depending on the specific cellular process. In fact, both BfmR and BfmS participate in modulation of motility by light, while only BfmR is involved in light regulation of desiccation tolerance in this microorganism. Neither BfmR nor BfmS contain a photoreceptor domain and then most likely, the system is sensing light indirectly. Intriguingly, this system inhibits *blsA* expression at 37 °C, suggesting antagonistic functioning of both signaling systems. Furthermore, we present evidence indicating that the phosphorylatable form of BfmR represses motility. Overall, we provide experimental evidence on a new biological function of this multifaceted system that broadens our understanding of *A. baumannii*’s physiology and responses to light.

## Introduction

We have extensively shown that *Acinetobacter baumannii* (*A. baumannii*) senses and responds to light. Particularly, we have accumulated a large amount of evidence about the light signal transduction and physiological responses at moderate temperatures such as 23 °C, which are mainly but not only governed by the blue light using FAD (BLUF)-type photoreceptor BlsA^1–11^. We have also shown that *A. baumannii* responds to light at 37 °C modulating virulence in an epithelial infection model using human keratinocytes in culture^11^, as well as *quorum sensing*^10^, through a BlsA-independent mechanism^10,11^. However, the light signal perception and transduction components operating at this temperature were not yet identified.

BfmRS is a two-component system (TCS) shown to control several pathways and physiological responses in *A. baumannii*^12^. The N-terminal receiver domain of the response regulator, BfmR, contains a well-conserved site of phosphorylation/activation, while the C-terminal effector domain contains a DNA-binding domain^13^. *bfmS* encodes a putative sensor kinase^14^, containing the pfam00672, cd00082 and cd00075 domains. These conserved regions represent the HAMP, the histidine kinase A, and the histidine-kinase-like ATPase domains, respectively, generally found in bacterial sensors^14,15^. Also, BfmS contains two membrane-spanning regions common to sensor kinases^12^, while the N-terminal extracellular region has no conservation to known sensors.

Recent results strongly suggest that phosphorylated BfmR (BfmR ~ P) is the active form of this response regulator that directly influences gene expression^12^. In addition, Palethorpe et al. have provided experimental evidence indicating that the BfmS sensor kinase acts as a BfmR phosphatase to negatively regulate BfmR activity in the studied conditions^12^. Moreover, it was shown that BfmR can autophosphorylate in vitro using small phosphodonors such as acetylphosphate^12^. Yet, the molecular mechanism of BfmRS functioning is far from being understood.

Extensive characterization of the BfmRS TCS by using single or double mutants, contributed to gain insights into the cellular processes governed in *A. baumannii*. In particular, BfmR has been shown to control pellicle and biofilm formation^14,16,17^. Further studies showed that BfmR is required for *A. baumannii* persistence in a murine lung infection model^18^, for growth in human ascites fluid and for serum resistance^19,20^. Moreover, it was shown to contribute to survival in a rat subcutaneous abscess model^21^, and in a neutropenic murine bacteremia model^22^. BfmR has been also shown to control tolerance to desiccation and responses to oxidative stress^12,23^.

BfmS supports increased tolerance to carbenicillin and fluoroquinolones, and controls production of outer membrane vesicles (OMVs) and OMV-mediated host cell cytotoxicity^17,20,24,25^. Also, adhesion to A549 cells and killing of bacteria in normal human serum were significantly reduced in the *bfmS* mutant, with only partial complementation of these phenotypes^17^. Furthermore, *bfmS* null strains were defective in growth/persistence in *Galleria mellonella* larvae^26^. In *A. nosocomialis* M2, the loss of BfmS results in a significant reduction of motility^27^.

The two-component system BfmRS has been also shown to be involved in negative regulation of the contact-dependent growth inhibition system (CDI)^16,28^, which modulates cell–cell and cell–environment interactions, potentially allowing bacteria to adapt to ever-changing conditions^16,28^. The evidence indicating down regulation of capsule gene expression or differential capsular polysaccharide profiles in *bfmRS* deletion is consistent in different publications^19,20,24,28^. Moreover, the *bfmRS* deletion presented hypersensitivity to a wide variety of antibiotics including β-lactams, rifampicin, erythromycin, ciprofloxacin and tobramycin^20^. Finally, BfmRS controls stress response, particularly oxidative and osmotic stress, at the level of the cell envelope^17,20,25^.

From analyses of ours as well as other researchers’ data^20^, we observed that the BfmRS system and light integrate signals into the same pathways in *A. baumannii.* In fact, BfmR/S and light regulate catalase production^3,23^; the phenylacetic acid degradation pathway^4,20^; the acetoin catabolic pathway^9^; trehalose biosynthesis; pellicle formation^4,16^, and biofilm formation^4,14^; as well as expression of the Acinetobactin operon^8^, genes coding for the AdeABC efflux pump^7,20^, genes coding for the efflux pump EmrAB^3,20^, components of the quorum sensing machinery such as *abaI*^10,20^, fimbria^3,20^ and the T6SS system^16^. Furthermore, it has been shown that BfmR interacts with BlsA mostly in the dark^29^. The overall evidence prompted us to study whether a connection exists between light modulation and the BfmRS system.

In this work, we show that both components of the BfmRS system are involved in light modulation of motility at 37 °C in *A. baumannii* V15. On the contrary, light modulates desiccation tolerance through a BfmR-dependent BfmS independent pathway, both at moderate (23 °C) as well as at the normal temperature in mammals (37 °C), in this microorganism. The overall data indicate that BfmRS is involved in light signal transduction in *A. baumannii*. Interestingly, this system inhibits *blsA* expression at 37 °C^1,2,4^, and 23 °C, although to a lesser extent in the latter. Neither BfmR nor BfmS contain a traditional photoreceptor domain, and thus the most plausible possibility is that the system is sensing light indirectly, most probably as a result of differential metabolism arising under blue light vs. dark conditions.

## Results

### The BfmRS system integrates a light signal into motility at 37 °C

**Figure 1:**
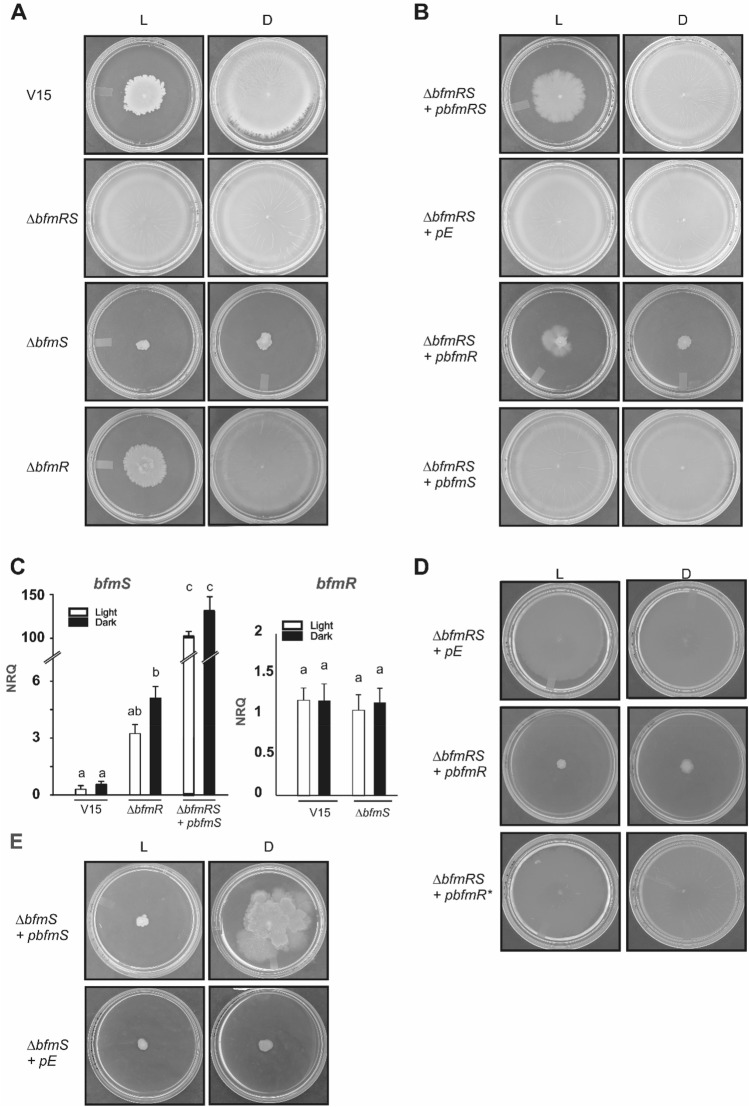
Effects of light and temperature on motility at 37 °C. (**A**) Cells of the parental strain *A. baumannii* V15 and the isogenic *ΔbfmRS*, *ΔbfmR* and *ΔbfmS* mutants were inoculated on the surface of motility plates. Plates were inspected and photographed after 18 h incubation in darkness (D) or in the presence of blue light (L) at 37 °C. (**B**) Cells of the V15 *ΔbfmRS* mutant transformed with the BfmRS, BfmR, or BfmS-complementing plasmids pBfmRS, pBfmR, or pBfmS, respectively; or the empty plasmid (pE), were inoculated on the surface of motility plates containing 0.1 mM IPTG. Plates were inspected and photographed after 18 h incubation in darkness (D) or in the presence of blue light (L) at 37 °C. (**C**) Estimation by qRT-PCR of the expression levels of *bfmS* in cells recovered from motility plates inoculated with *A. baumannii* V15, the *ΔbfmR* mutant and the *ΔbfmRS* mutant harboring the p*bfmS* plasmid (supplemented with 0.1 mM IPTG); incubated at 37 °C under blue light (L) or in the dark (D). Shown are the mean and standard deviation of normalized relative quantities (NRQ). Significant differences determined by ANOVA followed by Tukey’s multiple comparison test (*p* < 0.05) are indicated by different letters. Shown are representative results of three independent experiments. (**D**) Cells of the V15 *ΔbfmRS* mutant transformed with the BfmR and BfmR*-complementing plasmids pBfmR, and pBfmR*, respectively; or the empty plasmid (pE), were inoculated on the surface of motility plates containing 0.1 mM IPTG. Plates were inspected and photographed after 18 h incubation in darkness (D) or in the presence of blue light (L) at 37 °C. (**E**) Cells of the V15 *ΔbfmS* mutant transformed with the BfmS-complementing plasmid pBfmS, or the empty plasmid (pE), were inoculated on the surface of motility plates containing 0.1 mM IPTG. Plates were inspected and photographed after 18 h incubation in darkness (D) or in the presence of blue light (L) at 37 °C.

At 37 °C, *A. baumannii* V15 strain moved around the inoculation point on motility plates incubated overnight in the presence of blue light, while the bacteria covered the whole surface on plates incubated in the dark (Fig. 1A). Thus, light modulates motility in V15 strain at 37 °C. Interestingly, the *ΔbfmRS* mutant moved to a similar extent under blue light and in the dark at this temperature, indicating that the double mutant lost photoregulation of motility (Fig. 1A). Thus, the BfmRS system is involved directly or indirectly in light perception. On the contrary, the *ΔbfmS* mutant completely lost the ability to move at both conditions, showing loss of motility and photoregulation (Fig. 1A). Impairment of motility in the dark was also observed in the *A. nosocomialis* M2 *ΔbfmS* mutant by Clemmer et al.^27^. The *bfmR* mutant behaved similarly to the wild type strain (Fig. 1A). This was surprising since if BfmR had no role in the process, it would have been expected that the *ΔbfmRS* behaved as the *ΔbfmS* mutant. Besides, regulatory effects that result from the coordinated activity of BfmS and BfmR are expected to be similar in the *ΔbfmR* and *ΔbfmRS* mutant strains because both lack BfmR*.* This prompted us to speculate that in the absence of BfmR, BfmS might transduce the signal through a different cognate response regulator, as has been postulated for this system in a recent work^12^. In fact, it was shown that deletion of the response regulator can allow for amplification of cross-talk between the sensor kinase and other non-cognate response regulators, leading to indirect regulatory effects^12,30^. In this context and in agreement with our results, effects in the *ΔbfmR* mutant that are due to amplified cross-talk between BfmS and other regulators are not be observed in the *ΔbfmRS* mutant that lacks BfmS.

Complementation of *ΔbfmRS* mutant with pBfmRS, in which expression of the *bfmRS* operon is directed by the control of the inducible p*tac* promoter, rescued the wild type phenotype of regulation of motility by light (Fig. 1B). As control, the *ΔbfmRS* mutant harboring the empty pUC_AcORI_Ptac_TER_lacIq2 plasmid^16^, which is referred to as pE, behaved as *ΔbfmRS*, i.e., does not photoregulate and moved both under blue light and in the dark (Fig. 1B). In contrast, when *ΔbfmRS* was transformed with plasmid p*bfmR*, the bacteria lost ability to move (Fig. 1B), just as occurs with the *ΔbfmS* mutant, strongly suggesting a role for BfmR as a motility repressor in the absence of BfmS. When *ΔbfmRS* was transformed with p*bfmS*, the bacteria moved covering the whole plates both under light and dark conditions, behaving as it would be expected for a *ΔbfmR* mutant (Fig. 1B). The fact that this strain does not behave as actually does the *ΔbfmR* mutant, suggests that BfmS levels are finely tuned and determine the resulting phenotype. In fact, our qRT-PCR analyses show that *bfmS* levels are induced in the *ΔbfmR* mutant approximately 10 folds under both illumination conditions (Fig. 1C) indicating that, in addition to the absence of BfmR, additional changes at the regulatory level are taking place in this strain due to the missing BfmR-mediated regulation of the *bfmRS* operon (Fig. 1C). In this context, Palethorpe et al.^12^ showed that *bfmRS* transcriptional reporter levels were decreased in the *∆bfmR* mutant; also indicating BfmR-mediated regulation of *bfmRS* but acting as an activator rather than as a repressor^12^. *bfmS* levels in the *∆bfmRS* + p*bfmS* plasmid were more than 100 folds higher than those of the wild type both under blue light and in the dark. In this case, the sensor levels are so high that signal regardless of the illumination condition, a situation usually observed when regulators are expressed at high levels^4^. As expected, deletion of *∆bfmS* did not alter *bfmR* expression levels (Fig. 1C), consistent with absence of regulation of the *bfmRS* operon by BfmS^25^.

Moreover, *ΔbfmRS* was also transformed with plasmid pbfmR*, which expresses a mutant version of the *bfmR* allele leading to a substitution of the aspartate residue 58 to an alanine (D58A) in the corresponding protein. Aspartate 58 is the conserved phosphorylation site in the BfmR receiver domain^12,19^. Thus, mutation of this residue in BfmR prevents it from becoming activated by phosphorylation^12,13^. In this case, the bacteria moved covering the whole plates both under blue light and in the dark, indicating that it is the phosphorylatable version of BfmR which produces the inhibitory effect on motility (Fig. 1D).

Finally, the *ΔbfmS* mutant complemented with the plasmid expressing *bfmS* rescued motility in the dark at significant higher levels than under blue light, i.e., behaved similarly as the wild type (Fig. 1E). This indicates that restitution of BfmS restores photoregulation of motility and thus is directly involved in this response. Overall, these complementation results indicate that the effects observed are specific and the two-component system BfmRS transduces a light signal at 37 °C.

### *A. baumannii* V15 senses blue, green and red light at 37 °C

**Figure 2:**
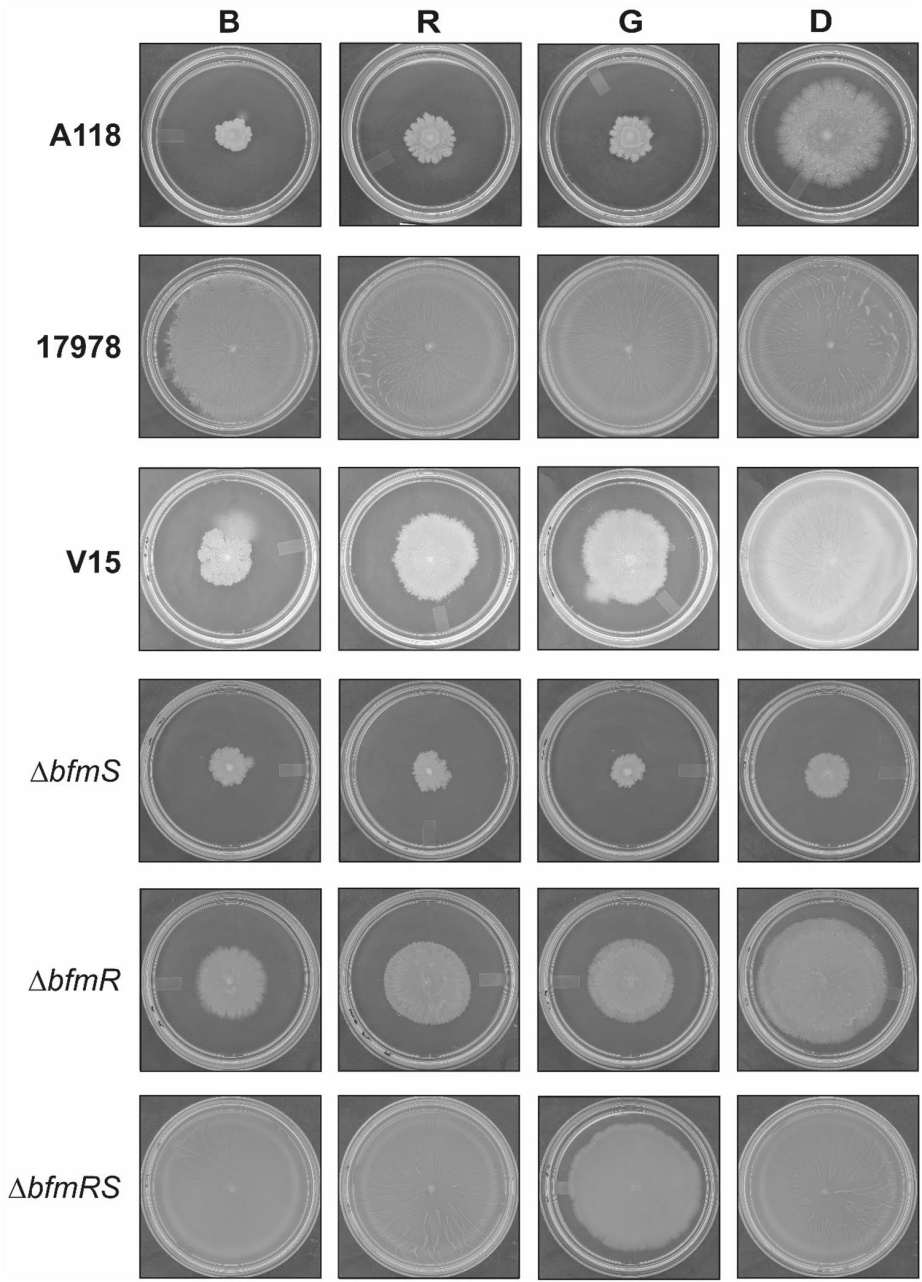
Effects of lights of different wavelengths on motility. Cells of the *A. baumannii* A118, ATCC 17978 and V15 strains, as well as the V15 isogenic *ΔbfmRS*, *ΔbfmR* and *ΔbfmS* mutants were inoculated on the surface of motility plates. Plates were inspected and photographed after 18 h incubation in darkness (D) or in the presence of blue (B), red (R) or green (G) light at 37 °C. Light was emitted by nine-LED (light-emitting diode) arrays with an intensity of 6 to 10 µmol photons/m^2^/s.

To further characterize the response to light, we evaluated the effect of light of different wavelengths on *A. baumannii* V15 and derived mutants’ motility at 37 °C. Interestingly, our results show that V15 not only senses blue, but can also respond to red and green light at this temperature (Fig. 2). In fact, V15 spread around the inoculation point under the different lights, while covered the whole plate in the dark. A similar pattern was observed in the case of strain A118 (Fig. 2), i.e., much higher motility in the dark respect to illumination conditions, indicating that the response to different lights is widespread at 37 °C and not restricted to V15 strain. In contrast, ATCC 17978 behaved differently, not responding to light at this temperature (Fig. 2). Thus, the overall results indicate that the ability to respond to light is strain-specific in *A. baumannii* strains, suggesting the presence of different light perception systems and/or signal transduction pathways.

Most interestingly, the V15 *ΔbfmRS* mutant completely lost the ability to sense any of the different light wavelengths (Fig. 2), implying that the BfmRS system is involved in sensing not only blue light but also in red and green wavelengths at 37 °C. The *ΔbfmS* strain did not move under red or green light, as it did not neither under blue light nor in the dark (Fig. 2). The *ΔbfmR* strain responded to red and green light in a similar fashion as to blue light, showing a marked reduction of motility with respect to dark conditions (Fig. 2). The *ΔbfmRS* strain does not sense either red or blue light, however it shows a slight response to green light (Fig. 2). Overall, the results show that the BfmRS system is involved, directly or indirectly, in red and green light sensing in *A. baumannii* V15 strain.

Comparisons of BfmR and BfmS protein sequences in *A. baumannii* strains ATCC 17978 (CP053098.1), A118 (GCF_000186565.1; AEOW00000000.1) and V15 (OP429427), showed that BfmR protein sequences are identical in the three strains analyzed (not shown). In turn, BfmS from V15 and 17978 have only one amino acid difference at position 455, in which a serine (S) changes for an asparagine (N), respectively (Supplementary Fig. 1). These represent two common variants within the *A. baumannii* BfmS population. The N455S difference lies within the histidine kinase domain, particularly in the Histidine kinase-like ATPase portion. Since the amino acidic difference lies in the kinase and not in the sensor domain, it is not likely that it is relevant for sensing light of different wavelengths. Comparison of BfmS protein sequences between V15 and A118 strains shows a Q to E substitution at position 166, an A to V substitution at position 492 and a T to S substitution at position 543 (Supplementary Fig. 1). Thus, there is no common pattern between A118 and V15 BfmS sequences respect to that of ATCC 17978, to explain the observed results. Most likely, differential ability to sense light of different wavelengths resides in other components of the light transduction machinery.

### BfmR represses *blsA* expression at 37 °C

**Figure 3:**
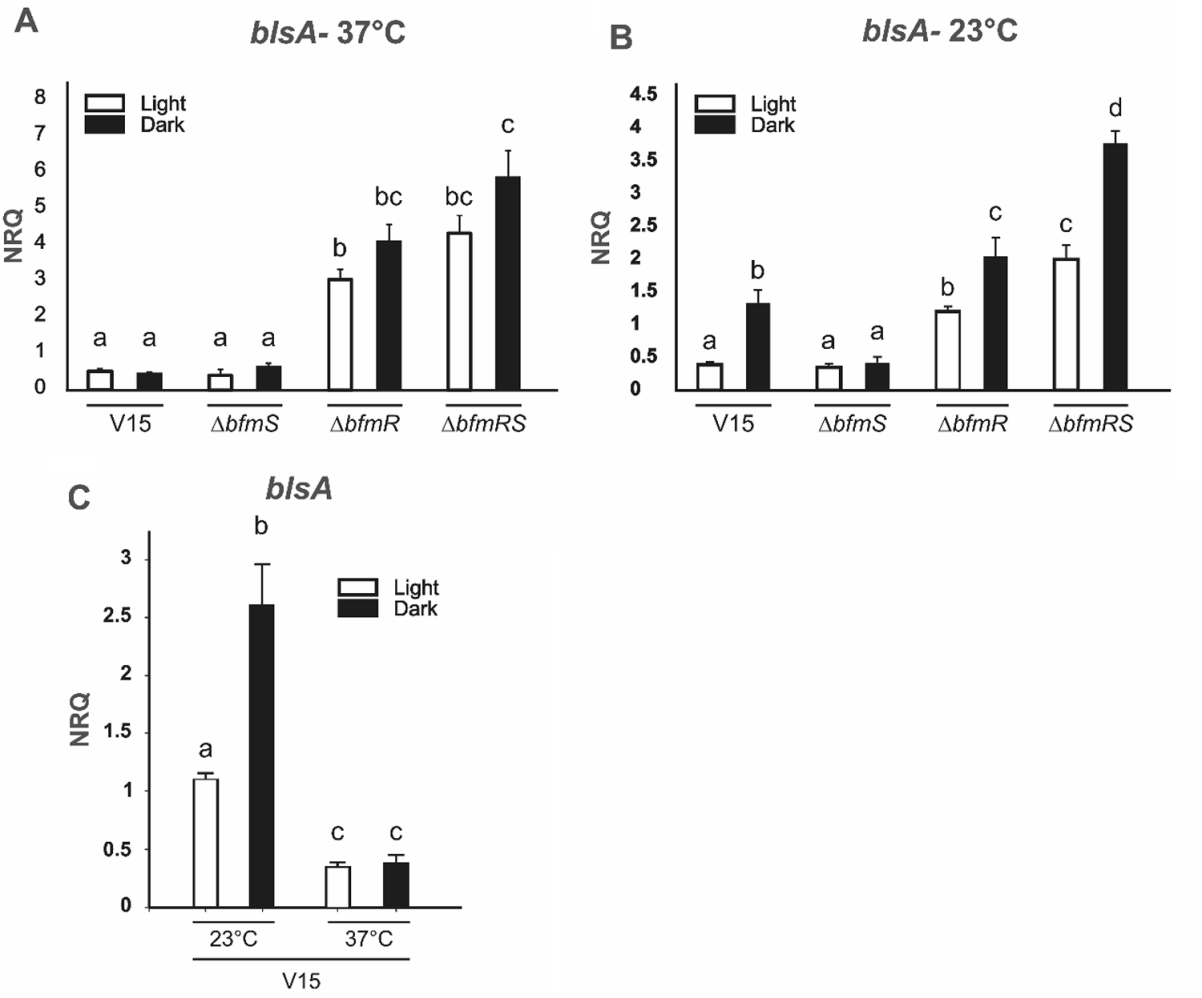
Effect of light and BfmRS on expression of *blsA*. Estimation by qRT-PCR of the expression levels of the gene coding for the photoreceptor *blsA*, in cells recovered from motility plates inoculated with *A. baumannii* V15 and derivative *ΔbfmRS*, *ΔbfmR* and *ΔbfmS* mutant strains incubated at 37 (**A**) or 23 °C (**B**) under blue light (L) or in the dark (D). In (**C**) are shown *blsA* expression levels in V15 at 37 °C compared to 23 °C, under blue light and in the dark. Shown are the mean and standard deviation of normalized relative quantities (NRQ). Significant differences determined by ANOVA followed by Tukey’s multiple comparison test (*p* < 0.05) are indicated by different letters. Shown are representative results of three independent experiments.

We next studied *blsA* expression in V15 and isogenic *bfmR/S* mutant cells recovered from motility plates incubated at 37 °C under blue light or in the dark. Our results show that *blsA* expression was significantly induced in *ΔbfmR* respect to the wild type under blue light and in the dark by approximately 6 and 8 folds, respectively; as well as 9 and 12 folds in the *ΔbfmRS* mutant respect to the wild type under blue light and in the dark, respectively (Fig. 3A). In contrast, *ΔbfmS* behaved similarly to the wild type (Fig. 3A)*.* Thus, the results are consistent with BfmR acting as a repressor of *blsA* expression at this temperature.

Interestingly, our data is consistent with analyses of RNA-seq data performed in ATCC 17978 published by Geisinger et al*.*, 2018, indicating that the fold change (FC) of expression of *blsA* at 37 °C (FC*blsA*) in *ΔbfmRS* respect to wild type (WT) is 4.91; FC*blsA* in *bfmR* respect to WT: 4.25 and FC*blsA* in *bfmS* to WT: 1.2^2,4^.

Overall, our results show that BfmRS controls *blsA* at the transcriptional level at 37 °C.

### BfmRS represses motility in *A. baumannii* V15 at moderate temperatures

**Figure 4:**
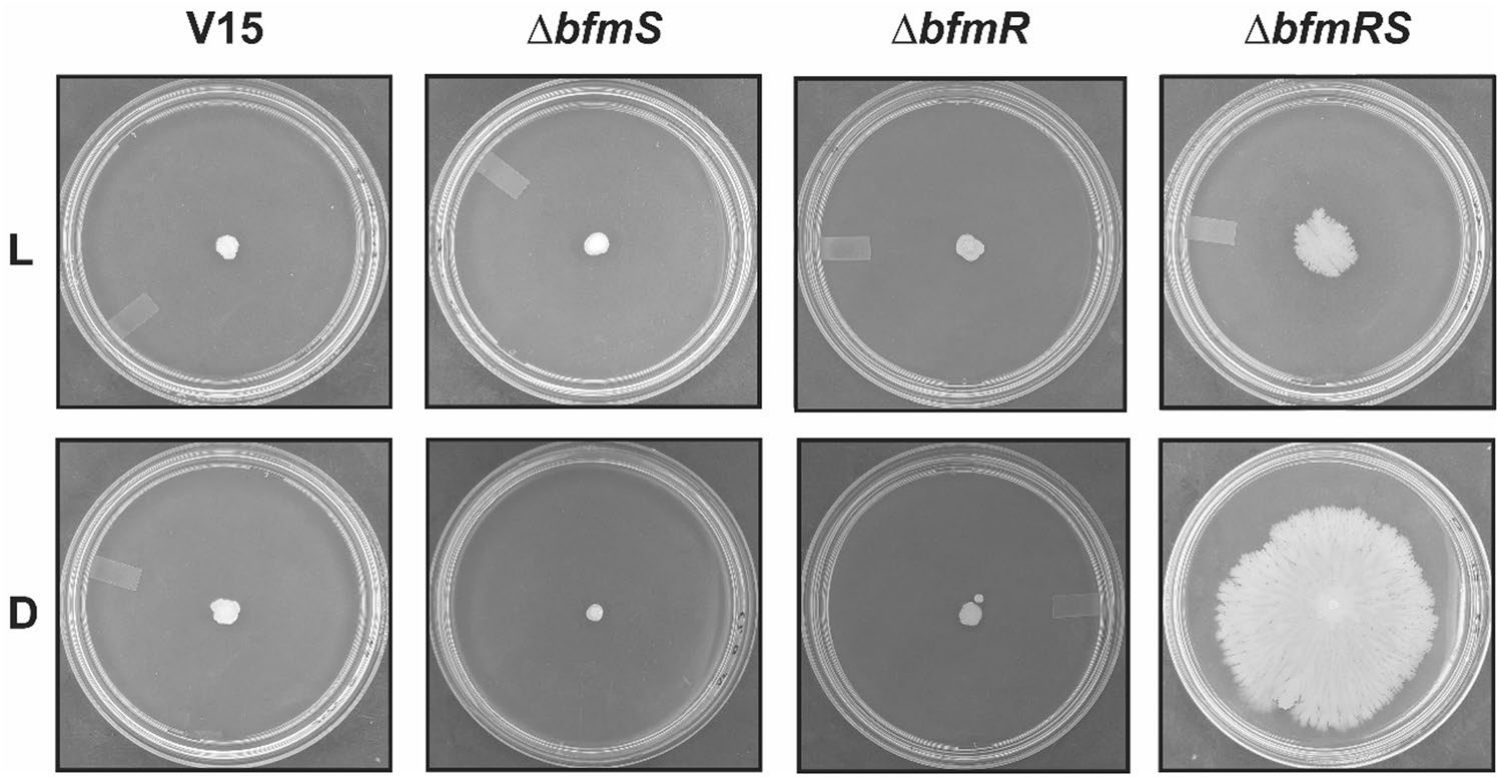
Effects of light and temperature on motility at 23 °C. Cells of the parental strain *A. baumannii* V15 and the isogenic *ΔbfmRS*, *ΔbfmR* and *ΔbfmS* mutants were inoculated on the surface of motility plates. Plates were inspected and photographed after 48 h incubation in darkness (D) or in the presence of blue light (L) at 23 °C.

We next examined motility in the wild type and isogenic mutants at moderate temperatures such as 23 °C, given that we have previously characterized responses to light in *A. baumannii* at this temperature^1–11^. Our results show that V15 as well as the *ΔbfmR* and *ΔbfmS* mutants do not move at this temperature, neither under blue light nor in the dark (Fig. 4). On the contrary, the *ΔbfmRS* mutant showed motility and regulation of motility by light at this temperature, which resembles BlsA-dependent phenotypes characterized previously for other strains at this temperature^4^.

In fact, at moderate temperatures *blsA* expression levels were induced approximately 4 and 2.6 folds in the *ΔbfmRS* mutant respect to the wild type under blue light and in the dark, respectively, when the cells were recovered from motility plates (Fig. 3B). In contrast to *blsA* expression at 37 °C, expression at 23 °C shows light-dependency, being induced approximately 3.5 folds in the dark respect to illuminated conditions (Fig. 3B). *blsA* levels were similar between the *ΔbfmS* mutant both under blue light and in the dark, and the wild type strain under blue light, while also showed induction in the *ΔbfmR* mutant, despite it was not as important as in the *ΔbfmRS* mutant (Fig. 3B). No correlation patterns are thus observed between motility and *bls*A expression in these strains; except for the higher *blsA* levels found in the *ΔbfmRS* mutant when incubated in the dark, a condition at which the strain is motile. Thus, it might be that a certain threshold of *blsA* levels should be reached to ensure motility. Furthermore, our qRT-PCR results show that *blsA* expression is modulated by temperature in V15 (Fig. 3C) since, *blsA* expression levels were induced at 23 respect to 37 °C both under blue light and in the dark, by approximately 3 and 6 folds, respectively. Thus, the observed *blsA* expression pattern is consistent with that previously observed in ATCC 17978^2,4^.

### BfmS does not participate in BfmR- light regulated desiccation tolerance

**Figure 5:**
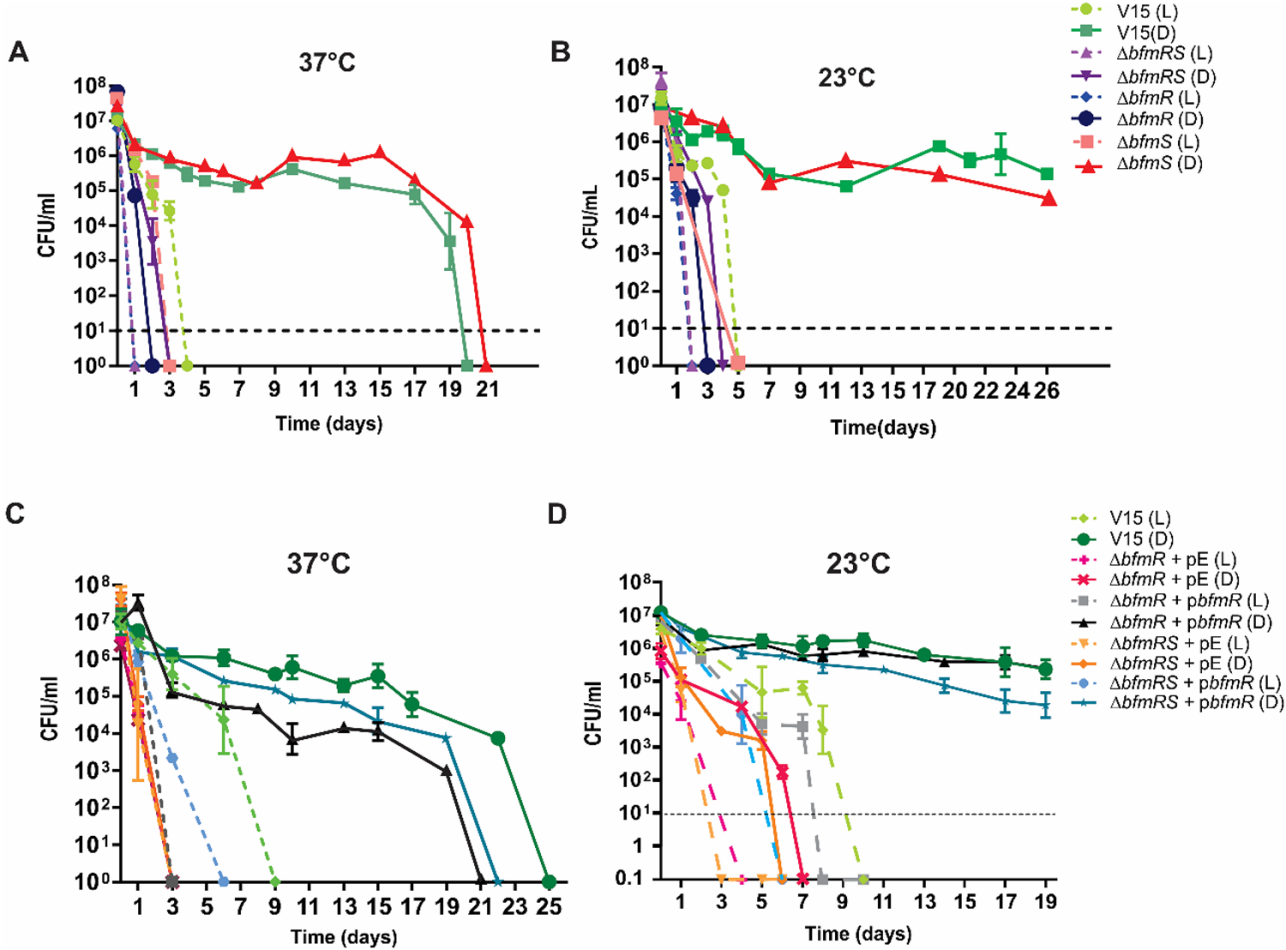
Light modulates desiccation tolerance through BfmR in *A. baumannii*. Dried cells of *A. baumannii* V15, as well as the *ΔbfmRS*, *ΔbfmR* and *ΔbfmS* mutants, placed on filter paper pieces were incubated under blue light (L) or in the dark (D) at 37 °C (**A**) or 23 °C (**B**). Cells of the *ΔbfmRS* or *ΔbfmR* mutants harboring the BfmR-complementing plasmid, pBfmR, or the empty plasmid, (pE), were grown in the presence of 0.1 mM IPTG, and then placed on filter paper pieces and incubated under blue light (L) or in the dark (D) at 37 °C (**C**) or 23 °C (**D**). Figure descriptions are the same for A and B and C and D panels and are indicated on the right of each pair of panels. Survival was assessed by determining the CFU counts per ml at the indicated times points. Shown is a representative result from at least three independent experiments. In each experiment three technical replicates for each strain and condition were included, and error bars indicate the standard error of the mean. In some cases, error bars are hidden by the point marker. Dotted lines represent the limit of detection, which corresponds to 10 CFU/ml in these experiments. All determinations below the limit of detection line received the arbitrary value of 0.1, for plotting purposes.

We further studied other phenotypes reported to be modulated by BfmR in *A. baumannii,* such as desiccation tolerance^23^. Desiccation tolerance has been shown to contribute directly to long term survival, which is a key feature in *Acinetobacter spp.*’s ability to spread in the hospital environment and between patients^31–33^. Moreover, we have shown that light modulates desiccation tolerance in *Staphylococcus aureus* and *Acinetobacter nosocomialis*^11^. Thus, we next evaluated the effect of light on *A. baumannii* V15 and isogenic *bfmR/S* mutants’ tolerance to desiccation, by inoculation of the bacteria on filter papers and subsequent incubation at 37 or 23ºC, under blue light or in the dark; after which the number of viable bacteria was determined at different time points. Our results show that V15 exhibits significantly higher desiccation tolerance in the dark compared to illumination conditions at both temperatures assayed (Fig. 5A and B). In fact, at 37 °C V15 cells remained relatively constant in number until day 17, decreasing approximately 1.5 logs at day 19, and reached undetectable levels by day 20. On the contrary, the number of viable bacteria under blue light decreased sharply from the beginning, falling below the limit of detection at day 4 (Fig. 5A). Thus, light modulates desiccation tolerance at 37 °C. The *ΔbfmR* and *ΔbfmRS* mutants showed significant susceptibility to desiccation both under blue light and in the dark, being much more susceptible than the wild type strain particularly in the dark (Fig. 5A). In fact, *ΔbfmR* and *ΔbfmRS* cells numbers fell below detection levels at day 1 under blue light, while were undetected at days 2 and 4 in the dark, respectively (Fig. 5A). Interestingly, the time lapse at which bacterial cells became undetectable between dark vs. light conditions in the *ΔbfmR* and the *ΔbfmRS* mutants, which was of 1 and 2 days, respectively, was significantly shorter than that of wild type strain (16 days); showing loss or significant reduction of photoregulation of dessication and thus indicating that BfmR participates in this process. Conversely, the *ΔbfmS* mutant behaved similarly to the wild type (Fig. 5A), indicating that BfmS does not participate in modulation of desiccation tolerance by light at this temperature. The behavior at 23 °C was similar to that described above for 37 °C (Fig. 5B). Complementation of *ΔbfmR* and *ΔbfmRS* with pBfmR rescued the responsiveness to light behaving like the wild type or the *ΔbfmS* strains, respectively, at both temperatures (Fig. 5C and D). The overall results thus show that light modulates resistance to desiccation and that this response depends on BfmR. Interestingly, no involvement of BfmS was detected in the conditions used in this study (Table 1).

**Table 1:** Primers used in this study.

Name	Sequence (5′–3′)	References
BfmR_F	GTTGaagcttAAATGCAGCAACATCTCC	This study
BfmR_R	GTTGaagcttCCATCAGTTAAAGATTCTCG	This study
D58A_mR	CAACATGACAGCCAAGACC	This study
D58A_mF	GGTCTTGGCTGTCATGTTG	This study
BfmR_Ptac_F	CATGAGCCAAGAAGAAAAGTTACC	This study
BfmR_Ptac_R	TTACAATCCATTGGTTTCTTTAAC	This study
*bfmS*F	AAATCCGACAGGTGCGTTATGC	^3^
*bfmSR*	ATACGTGCCACAGGTGTTCTGA	^3^
*bfmRF*	CGATGGTAACCGTGCAATTCGT	^3^
*bfmRR*	ATCGTCTGCACCCATTTCCAGA	^3^
*rpoBF.rt*	CAGAAGTCACGCGAAGTTGAAGGT	^3^
*rpoBR.rt*	AACAGCACGCTCAACACGAACT	^3^
*recAF.rt*	TACAGAAAGCTGGTGCATGG	^4^
*recAR.rt*	TGCACCATTTGTGCCTGTAG	^4^

### Susceptibility to ampicillin is mediated by BfmR but is not modulated by light or BfmS

The involvement of BfmRS in antibiotic susceptibility has been reported by different groups^17,20,25^, obtaining in some cases opposite results. In this work, we examined susceptibility to ampicillin under blue light or in the dark at 37 °C in LB. Our results show that the mutants *ΔbfmRS* and *ΔbfmR* display hypersensitivity to ampicillin, with MIC values of 4 and 8 µg/ml, respectively, while the wild type strain V15 exhibits MIC values of 32 µg/ml (Table 2). Interestingly, the *ΔbfmS* mutant behaved as the wild type, indicating that BfmS does not participate in susceptibility to this antibiotic. It should be noted that the strains behaved similarly under blue light or in the dark, with no observable effect of light. Thus, BfmR but not BfmS is involved in ampicillin resistance, and light does not participate in this process. Overall, the BfmRS system transduces a light signal selectively depending on the cellular process.

**Table 2:** Susceptibility of *A. baumannii* mutants and their parental strains to ampicillin at 37 °C.

Antibiotic	MIC (µg/ml)			
	V15	*bfmRS*	*bfmS*	*bfmR*
**Ampicillin**				
L	32	4	32	8
D	32	4	32	8

## Discussion

**Figure 6:**
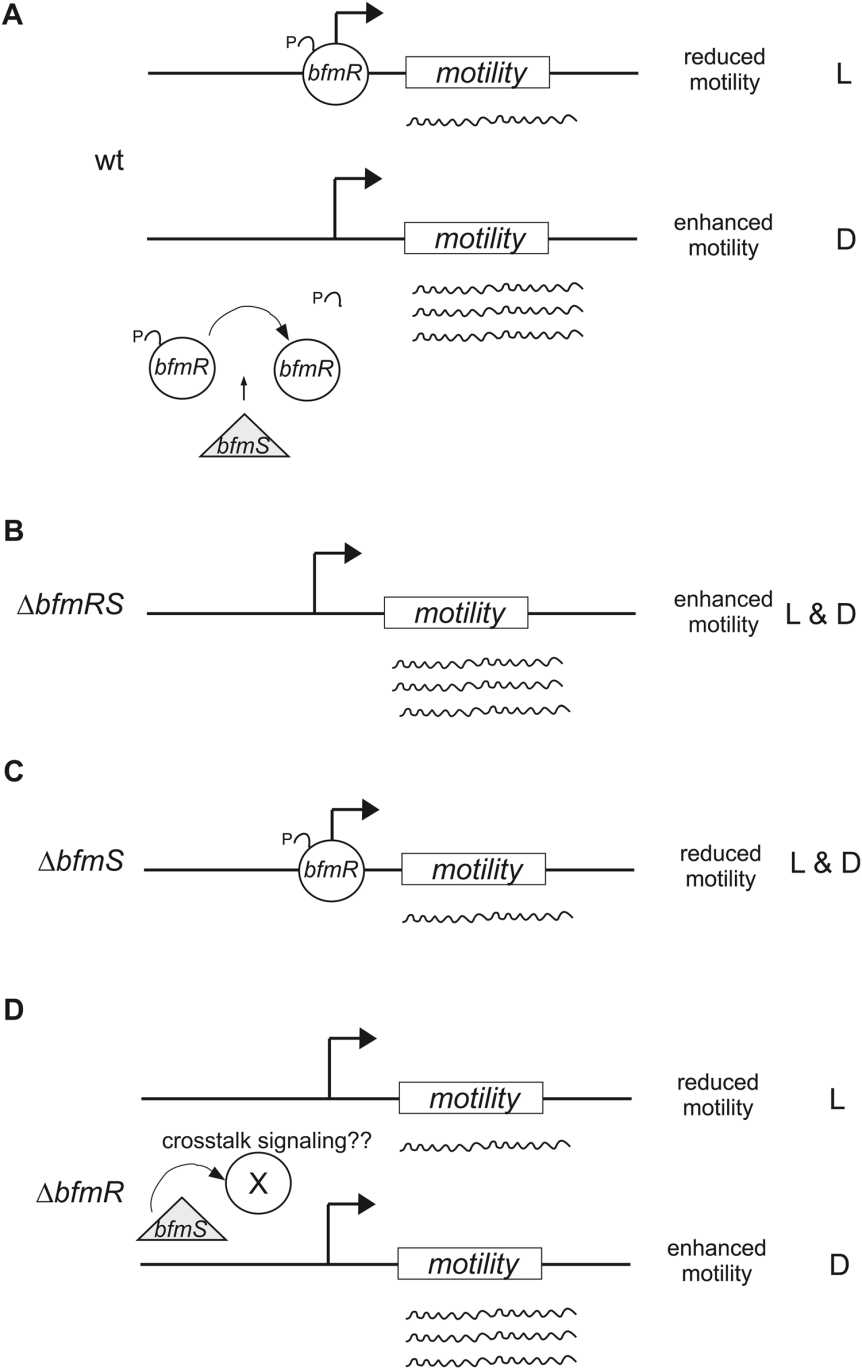
Working model depicting BfmRS involvement in light signaling of motility at 37 °C in *A. baumannii* V15 (wt: wild type). BfmR acts as a repressor of motility most likely in its phosphorylated state, and its functioning is counteracted by BfmS in the dark most probably by dephosphorylation, setting thus a differential response to illumination (**A**), which is lost in the *ΔbfmRS* mutant independently of the light condition (**B**). In this sense, the *ΔbfmS* mutant shows reduced or null motility both under blue light and in the dark (**C**), consistent with the repressor being active in its phosphorylated state. BfmS is thus transducing a light signal into this process, most probably as a result of increased dephosphorylation activity in the dark compared to light conditions. The signals leading to BfmS’s differential activity under blue light and in the dark are still unknown. Also represented is the case of *ΔbfmR* strain (**D**)*,* which is complex due to additional changes at the regulatory level occurring in *bfmS* expression as a result of BfmR’ absence, which likely result in amplification of signaling crosstalk. As the genes or gene pathways involved in motility in V15 at 37 °C are not unknown, they are represented here as a box named “motility”. L: light; D: dark.

The BfmRS two component system has been extensively characterized and shown to display atypical characteristics, despite the signals involved were still unidentified. In this work, we present evidence indicating that the BfmRS system transduces a light signal at 37 °C. In fact, we show that modulation of motility by light in *A. baumannii* V15 depends on both components of this system, BfmR and BfmS. Our working model is compatible with BfmR acting as a repressor of motility in its phosphorylated state, which is counteracted by BfmS in the dark most probably by dephosphorylation^12^, setting thus a differential response to illumination that is lost in the absence of the repressor, independently of the light condition (Fig. 6). Thus, our evidence indicates that BfmS is transducing a light signal into the process of motility, probably as a result of increased dephosphorylation activity in the dark compared to light conditions (Fig. 6). However, it does not seem to be the photoreceptor per se as it does not contain a light sensing domain. Most likely, it integrates light signals indirectly, which may arise from differential metabolism in light vs. dark conditions. The possibility also exists that photoreceptors encoded in the V15 genome would account for light perception and then transfer this information to BfmRS. In this sense, we would not expect the only traditional photoreceptor encoded the genome, BlsA, to be involved in this process, since we have previously shown that it only operates at moderate temperatures in *A. baumannii* ATCC 17978^1,2,4,34^. In fact, *blsA* expression is significantly lower at 37 °C than at 24 °C in *A. baumannii* ATCC 17978; and BlsA levels cannot be detected at temperatures equal or higher to 26 °C^2,4^. In this work, we show that expression of *blsA* is repressed by BfmRS in V15 at 37 °C, and further studies will contribute to ascertain whether BlsA is non-functional at 37 °C in this strain either^2,4^. Another gene encoded in the *A. baumannii* genome that could account for photoreceptor activity is one putative photolyase/cryptochrome, however, its physiological role has not been yet ascertained. Moreover, the existence of GAF-type photoreceptors that sense blue, red and green light have been reported *Nostoc flagelliforme*^35^. The possibility thus exists that there is/are new undescribed photoreceptor/s with this wide light perception range encoded in *A. baumannii* V15.

At moderate temperatures such as 23 °C, photoregulation of motility in V15 occurs only in the *ΔbfmRS* mutant, showing an inhibitory effect of BfmRS in this cellular process. In this case, the induction of *blsA* expression both under blue light and in the dark in the *ΔbfmRS* mutant as well as the *ΔbfmR* mutant, shows an inhibitory role for BfmR which is higher in the dark than in the presence of light, consistent with previous results published by Chitrakar et al.^29^. In this work, an interaction between BlsA and BfmR was determined by immunoprecipitation studies^29^, which was tighter in the dark respect to illumination conditions^29^. Overall, both BlsA and BfmRS seem to be operating at moderate temperatures, despite an inhibitory effect of BfmRS on BlsA is observed; while at 37 °C BfmRS inhibits *blsA* expression. It is possible though that BfmR binding to BlsA precludes its functioning and stimulation of its own synthesis. BfmS is not always involved in BfmR-regulated cellular pathways, for example in desiccation tolerance. This has been previously reported, for example, it has been shown that BfmR is essential for cell attachment and initiation of biofilm formation in plastic in strain ATCC 19,606, while BfmS has a dispensable role in this phenotype. In addition, the disruption of *bfmS* in the Bfm273 insertion derivative did not abolish the production of CsuAB^14^. The involvement of BfmR in the response to light observed in desiccation tolerance, which is BfmS independent, suggests that directly or indirectly BfmR is able to perceive light. One plausible explanation is that some unknown photoreceptor or alternative signal sensor would perceive the illumination and change the phosphorylation status of BfmR (or other posttranslational modification), transducing thus a light signal both at 23 and 37 °C. Finally, there are other phenotypes such as antibiotic susceptibility to ampicillin, in which BfmR is clearly involved, despite no effect of illumination is observed, which suggest that the mechanism of signaling through BfmRS is complex, involving multiple actors that respond differently to similar signals.

We further show that the phosphorylatable form of BfmR represses motility in *A. baumannii* V15. Our evidence indicating that *bfmS* levels are higher in a *∆bfmR* mutant respect to the wild type suggest BfmR acts as a repressor of the *bfmRS* operon in the studied conditions, contrasting a previously proposed role as activator^12,13^. These differences may be attributed to strain-specific effects and/or to differences in the culture conditions. In this context, the possibility exists that the different phosphorylated forms of BfmR (phosphorylated vs. dephosphorylated) function activating or repressing the *bfmRS* operon, depending on the conditions prevailing in the environment. In fact, this situation has been previously described for other response regulators such as HnoC in the *Shewanella oneidensis* nitric oxid signaling network^36^. Specifically, in the unphosphorylated state HnoC forms a tetramer, which tightly binds to an inverted-repeat target sequence overlapping with the promoter regions and repressing transcription. Phosphorylation of HnoC induces dissociation of the response regulator tetramer and detachment of subunits from the promoter DNA, which subsequently leads to transcriptional derepression^36^.

This work contributes significantly to our understanding of light regulation and BfmRS functioning, and also rises several questions regarding for example which are the genes responsive to light governing motility at 37 °C, given that the type I pilus (*prp* system) does not respond to light at that temperature shown in strain ATCC 17978 by Wood et al.^37^, which is in agreement with our qRT-PCRs performed in V15 cells recovered from motility plates under blue light vs. dark at 37 °C (Supplementary Fig. 2). Besides, expression of *pilT,* a component of type IV pili shown to be involved in twitching motility^27^, is neither responsive to light in V15 under the same conditions (Supplementary Fig. 2). Other important question that underlies is what detailed molecular mechanism and components are involved in signaling through BfmRS from light perception to the physiological responses, which involves selective responses and fine tuning of regulator levels, and which also may imply fine tuning of phosphorylation/dephosphorylation activities. All these aspects are currently being investigated in our laboratory.

## Methods

### Bacterial strains, plasmids, and media

The bacterial strains and plasmids used in this work include *A. baumannii* V15 and its derived isogenic mutants *ΔbfmR*, *ΔbfmS* and *ΔbfmRS* as well as the different complementing plasmids, which proceed from Krasauskas et al.^16^. In addition, *A. baumannii* type strain ATCC 17978 as well as A118^38^ were also used in this study. Luria–Bertani (LB) broth (Difco) and agar (Difco), as well as tryptone media (tryptone 1%, NaCl 0.5%, agarose 0.3%) were used to grow bacterial strains. Broth cultures were incubated either statically or with shaking at 200 rpm at the indicated temperatures.

### Construction of pBfmR*

The inducible plasmid with the mutant *bfmR* (D58A) allele was generated as follows: the beginning and ending of the *bfmR* gene was amplified with primer pairs BfmR_F/BfmR_D58A_mR and BfmR_D58A_mF/BfmR_R (Table 1), respectively. The fragment was joined by the overlap extension PCR using primer pair BfmR_Ptac_F/BfmR_Ptac_R and blunt ligated into previously prepared plasmid pUC_AcORI_Ptac_gfp_TER_lacIq2 as described in Krasauskas et al.^16^.

### Blue light treatments

Blue light treatments were performed as described in our previous studies^1–11^. Briefly, cells were incubated for 24 h (or else as specified) at 37 °C in the dark or under blue light emitted by 9-light-emitting diode (LED) arrays, with an intensity of 6 to 10 µmol photons/m^*2*^/s. Each array was built using three-LED module strips emitting blue, green, or red light with emission peaks centered at 462 nm, 551 nm, and 633 nm, respectively, as determined using a Ocean Optics (Flame) spectroradiometer^4^.

### Cell motility experiments

Motility plates containing 1% tryptone, 0.5% NaCl and 0.3% agarose^4^ were used as a tool to detect cell motility on a semisolid surface. The plates were inoculated on the surface with bacteria lifted from overnight LB agar cultures using flat-ended sterile wooden sticks or depositing 0.003 ml of LB cultures grown to an optical density at 600 nm (OD_600_) of 0.3. Plates were incubated for 18 h (overnight) or 48 h at 37 °C or 23 °C, respectively, in the dark or under light emitted by nine-LED (light-emitting diode) arrays with an intensity of 6 to 10 µmol photons/m^2^/s. Each array was built using three-LED module strips emitting blue, green, or red light with emission peaks centered at 462 nm, 514 nm, and 636 nm, respectively, as determined using a LI-COR LI-1800 spectroradiometer (see Fig. S1B in the supplemental material)^4^.

### Analyses of gene expression by qRT-PCR

Reverse transcription and qRT-PCR analyses were done as described in Tuttobene et al.^8^, using primers listed in Table 1 and Müller et al.^3^. Data are presented as NRQ (Normalized relative quantities) calculated by the qBASE method^39^, using *recA* and *rpoB* genes as normalizers.

### Desiccation assay

*A. baumannii* strain V15 and derived mutants were grown overnight in 10 ml of LB at 23 or 37 °C under blue light or in the dark. Cells in stationary phase were centrifuged (5000 rpm) and resuspended in sterile 0.9% NaCl. The bacterial concentration was adjusted to inoculate approximately 1 × 10^7^ CFU on each sample. After 3 h starvation, 20 µl of cell suspensions were spotted onto sterile cellulose filter paper pieces (whatman 1.5 cm × 1.5 cm), which had been previously sterilized by autoclaving. The membrane filters were placed in sterile Petri dishes and incubated at 23 or 37 °C under blue light or in the dark. At each selected time point, filter paper pieces were placed in tubes containing 1 ml of LB, which were subsequently vortexed vigorously for 10 s. Vortexing was repeated after a 20 min incubation at 37 °C and 200 rpm to remove the bacteria from the filter. Serial dilutions were prepared on 0.9% NaCl and plated on LB agar plates, and then incubated at 37 °C for 24 h to finally determine CFU/ml. Three technical replicates were analyzed at each time point studied, and three independent experiments were performed.

### MIC determination in liquid medium

MIC determination was performed in multi-well microplates using LB broth at 37 °C. The antibiotic ampicillin was subjected to serial half dilutions starting from 1024 µg/ml. The strains to be tested were resuspended in physiological solution and adjusted to OD_600_ 0.1, then diluted 1:10 in LB medium and applied to the wells. Identical microplates were incubated overnight in the dark or under blue light at 37 °C.

## Supplementary Information

Supplementary Information.

## Data Availability

All data generated or analyzed during this study are included in this published article.
