# Supplementary material for: BfmRS encodes a regulatory system involved in light signal transduction modulating motility and desiccation tolerance in the human pathogen Acinetobacter baumannii
Source: Sci Rep. 2023 Jan 5;13:175. doi: 10.1038/s41598-022-26314-8 (PMC9814549; doi:10.1038/s41598-022-26314-8)
Supplement: Supplementary file 1 — Supplementary Information. [file 41598_2022_26314_MOESM1_ESM.pdf]

Supplementary Figure 1

```
V15        VFKHSIFLRIYAGLVILVVLVAVFGYLLVQIINYQRAQEYRESLTDGISYVISEGVARQP
17978      VFKHSIFLRIYAGLVILVVLVAVFGYLLVQIINYQRAQEYRESLTDGISYVISEGVARQP
A118       VFKHSIFLRIYAGLVILVVLVAVFGYLLVQIINYQRAQEYRESLTDGISYVISEGVARQP
           ************************************************************

V15        GKQQKIDWISDASDLLELPIYYTDASKVELSRTEKKRIEAQKSVVRYDASNSIAYVIIGL
17978      GKQQKIDWISDASDLLELPIYYTDASKVELSRTEKKRIEAQKSVVRYDASNSIAYVIIGL
A118       GKQQKIDWISDASDLLELPIYYTDASKVELSRTEKKRIEAQKSVVRYDASNSIAYVIIGL
           ************************************************************

                                                        166
V15        RDDPQHYLSIKVDKITERQMKALPIFVLDYLMFYPGQEQEYLAKIQKHFSYPINIQNIQD
17978      RDDPQHYLSIKVDKITERQMKALPIFVLDYLMFYPGQEQEYLAKIQKHFSYPINIQNIQD
A118       RDDPQHYLSIKVDKITERQMKALPIFVLDYLMFYPGQEQEYLAKIEKHFSYPINIQNIQD
           *********************************************:**************

V15        VNLDSEQIGRLRQDQSVMLYKDSATVRGTTISIVSPIPNHPAQVLVLGPVPMFNWMPLQL
17978      VNLDSEQIGRLRQDQSVMLYKDSATVRGTTISIVSPIPNHPAQVLVLGPVPMFNWMPLQL
A118       VNLDSEQIGRLRQDQSVMLYKDSATVRGTTISIVSPIPNHPAQVLVLGPVPMFNWMPLQL
           ************************************************************

V15        SAGITLFSLFLLSLGVYGLILPLERKIRQVRYALNRMKSGDLSLRVPIEGSDEMANLASS
17978      SAGITLFSLFLLSLGVYGLILPLERKIRQVRYALNRMKSGDLSLRVPIEGSDEMANLASS
A118       SAGITLFSLFLLSLGVYGLILPLERKIRQVRYALNRMKSGDLSLRVPIEGSDEMANLASS
           ************************************************************

V15        YNNMSDHIQRLIEAQRELMRAVSHELRTPVARIRFGTEMLAEEDDYNHRMHQVDMIDKDI
17978      YNNMSDHIQRLIEAQRELMRAVSHELRTPVARIRFGTEMLAEEDDYNHRMHQVDMIDKDI
A118       YNNMSDHIQRLIEAQRELMRAVSHELRTPVARIRFGTEMLAEEDDYNHRMHQVDMIDKDI
           ************************************************************

V15        EALNTLIDEIMTYAKLEQGTPSLDFAEIVLFEVLDQVAVETEALKTQKEIELIPPPLYVK
17978      EALNTLIDEIMTYAKLEQGTPSLDFAEIVLFEVLDQVAVETEALKTQKEIELIPPPLYVK
A118       EALNTLIDEIMTYAKLEQGTPSLDFAEIVLFEVLDQVAVETEALKTQKEIELIPPPLYVK
           ************************************************************

                                         455
V15        VDAERRYLHRVVQNLVGNAVRYCDNKVRITGGIHSDGMAFVCVEDDGPGIPEQDRKRVFE
17978      VDAERRYLHRVVQNLVGNAVRYCDNKVRITGGIHNDGMAFVCVEDDGPGIPEQDRKRVFE
A118       VDAERRYLHRVVQNLVGNAVRYCDNKVRITGGIHSDGMAFVCVEDDGPGIPEQDRKRVFE
           **********************************.*************************

                     492
V15        AFARLDDSRTRASGGYGLGLSIVSRIAYWFGGEIKVDESPSLGGARFIMTWPAHRFKQPP
17978      AFARLDDSRTRASGGYGLGLSIVSRIAYWFGGEIKVDESPSLGGARFIMTWPAHRFKQPP
A118       AFARLDDSRTRVSGGYGLGLSIVSRIAYWFGGEIKVDESPSLGGARFIMTWPAHRFKQPP
           ***********.************************************************

            543
V15        LKTNKKAPA
17978      LKTNKKAPA
A118       LKSNKKAPA
           **:******
```

Supplementary Figure 1. Amino acid alignment of BfmS for the three *A. baumannii* strains V15, ATCC 17978 and V15. The numbers above the sequences indicate positions in the alignments where differences reside. In green are indicated differences between V15 and A118 respect to ATCC 17978, while

in blue are indicated differences between V15 and ATCC 17978 respect to A118. In pink boxes are indicated the predicted membrane spanning regions estimated by SOSUI (https://harrier.nagahama-i-bio.ac.jp/sosui/), which flank the extracellular sensor domain; and the C-terminal domain contains the histidine kinase domain. Identical (*) and conserved (:) amino acids at the given position are indicated below alignments.

Supplementary Figure 2

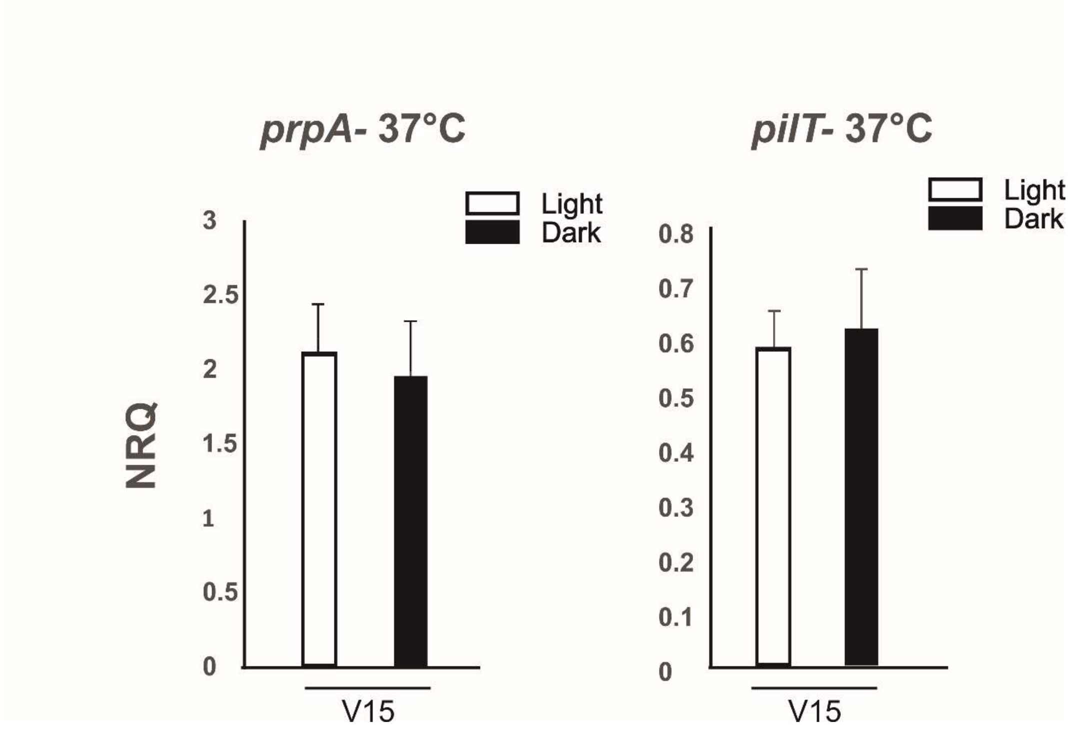

Supplementary Figure 2. Estimation by qRT-PCR of the expression levels of *prpA* and *pilT* in V15 cells recovered from motility plates incubated under blue light (L) or in the dark (D) at 37ºC. The data shown are mean ± SD of normalized relative quantities (NRQs) calculated from transcript levels measured in at least

three biological replicates.
